# Acute Tubular Necrosis after Ingestion of a Fertilizer Containing Sodium Silicate

**DOI:** 10.1155/2014/792954

**Published:** 2014-12-10

**Authors:** Hyeon Woo Lee, Yong Jun Choi, Se Won Oh, Hye Kyeong Park, Kum Hyun Han, Han Seong Kim, Sang Youb Han

**Affiliations:** ^1^Department of Internal Medicine, Inje University Ilsan-Paik Hospital, Joowha-ro 170, Ilsanseo-gu, Goyang, Gyeonggi 411-706, Republic of Korea; ^2^Department of Pathology, Inje University Ilsan-Paik Hospital, Joowha-ro 170, Ilsanseo-gu, Goyang, Gyeonggi 411-706, Republic of Korea

## Abstract

Silica nephropathy occurs after chronic heavy exposure to silica, resulting in the development of chronic kidney disease and progression to end-stage renal disease. However, acute kidney injury due to silica exposure is rare and its renal pathology remains unclear. Here, we report a case of acute sodium silica poisoning presenting as acute kidney injury. A 42-year-old man ingested a fertilizer containing sodium silicate. His serum creatinine increased by 5.06 mg/dL from 1.1 mg/dL 2 days after silicate ingestion. Owing to the decline in kidney function despite fluid therapy, a kidney biopsy was performed. The kidney showed acute tubular necrosis without infiltration of inflammatory cells. On day 5 of admission, hemodialysis was initiated to treat the hyperkalemia and oliguria, and treatment with methylprednisolone was initiated for the acute lung injury. The patient was administered 1 mg/kg of methylprednisolone intravenously daily for 2 weeks, followed by a 2-week taper. Hemodialysis was discontinued on day 10 and the patient's renal function recovered completely. However, he died on day 40 of hospitalization owing to complicated lung fibrosis and persistent pneumothorax/pneumomediastinum.

## 1. Introduction

Silicate-induced lung diseases such as pulmonary fibrosis are well known to occur in workers with long-term exposure to silica, such as miners, ceramic workers, and glass manufacturers [1]. Silica nephropathy also occurs after chronic heavy silica exposure, resulting in the development of chronic kidney disease (CKD) and progression to end-stage renal disease (ESRD) [2]. However, acute kidney injury (AKI) due to silica exposure is rare and its renal pathology remains unclear. Here, we report a case of acute sodium silica poisoning presenting as AKI.

## 2. Case

A 42-year-old man was referred to our hospital for treatment after intentionally ingesting fertilizer containing 100% sodium silicate. He had ingested about 50 mL of fertilizer 2 days prior to admission. On presentation, he complained of a sore throat and epigastric pain. The patient had a medical history of unmedicated chronic hepatitis B and had worked in a car-manufacturing factory for 10 years. He had a 20 pack-year smoking history. On admission, his blood pressure was 130/78 mmHg, pulse rate was 98 beats per minute, respiratory rate was 22 breaths per minute, and body temperature was 38.3°C. He had mild painful oral erosion and his lungs sounded clear to auscultation. Laboratory results from another hospital immediately after the ingestion had revealed the following findings: serum creatinine (Cr), 1.1 mg/dL; white blood cell (WBC) count, 113,600 cells/*μ*L; the arterial blood gas analysis (ABGA) results were as follows: pH, 7.340; PCO_2_, 40.3 mmHg; PO_2_, 96.8 mmHg; HCO_3_, 21.2 mm/L; and O_2_ saturation, 96.8% on room air. On admission to our hospital, laboratory findings were as follows: hemoglobin, 16.3 g/dL; WBC count, 23,290 cells/*μ*L; platelet count, 167,000 cells/*μ*L; blood urea nitrogen (BUN), 48 mg/dL; serum Cr, 5.06 mg/dL; total bilirubin, 1.7 mg/dL; aspartate aminotransferase, 68 U/L; alanine aminotransferase, 43 U/L; and glucose, 140 mg/dL. ABGA results were as follows: pH, 7.415; PCO_2_, 36.6 mmHg; PO_2_, 73 mmHg; HCO_3_, 25.5 mm/L; and O_2_ saturation, 95% on room air. Urinary analysis revealed a pH of 6.0, specific gravity of 1.010, 2+ protein, 2+ glucose, trace occult blood, and no WBCs, with proteinuria at 550 mg/day. Antinuclear antibody (ANA) titers were below 1 : 40 (nuclear pattern) and results for other serologic markers were nonspecific. Abdominal sonography revealed increased kidney size with increased parenchymal echogenicity.

**Figure 1:**
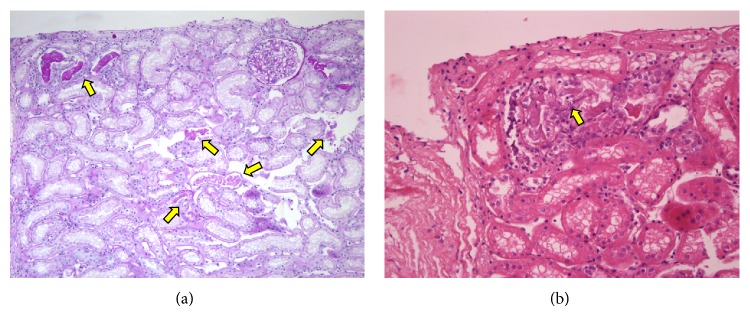
Light microscopic findings. (a) Low power view reveals break downs of tubules due to tubular cast (arrow; PAS ×100). (b) Light microscopic findings show necrotic and sloughed tubular cells in the tubular lumina. Regenerating tubular epithelial cells are noted with mitotic figures (arrow; hematoxylin and eosin ×200).

**Figure 2:**
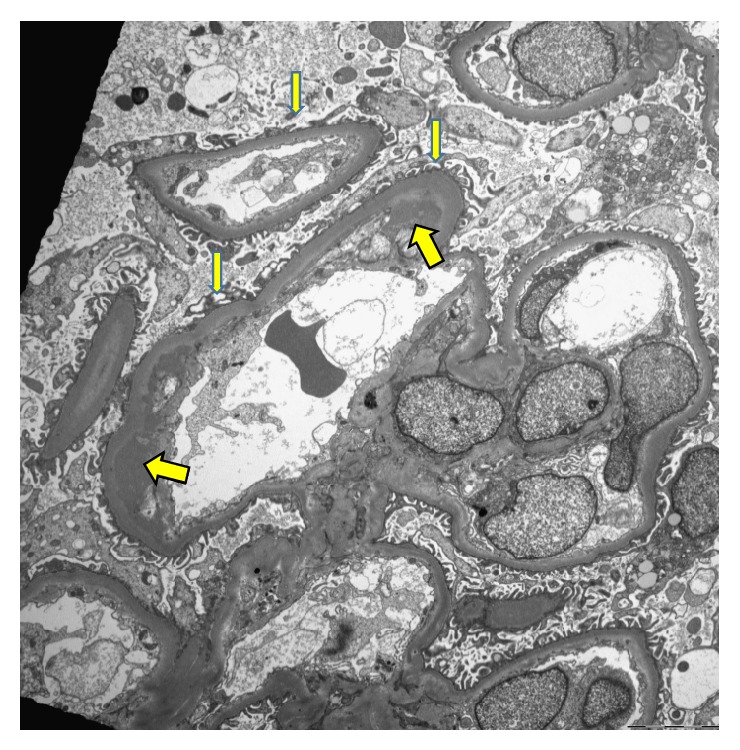
Ultrastructural examination reveals subendothelial electron-dense materials (thick arrows). Focal loss and fusion of foot processes of podocytes are also noted (thin arrows) (TEM ×2500).

**Figure 3:**
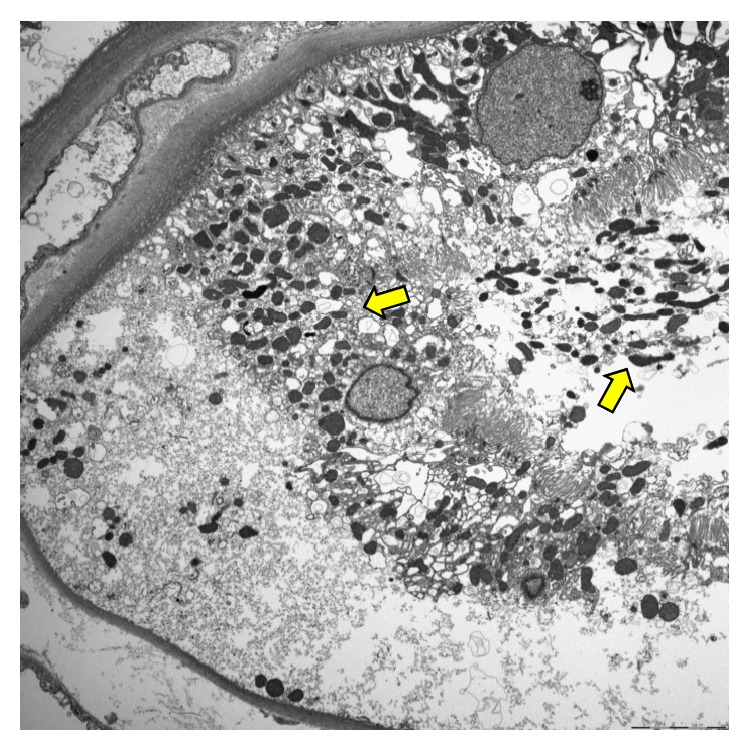
Ultrastructural examination of proximal tubules shows electron-dense lysosomes in both cytoplasm and tubular lumina (thick arrows) (TEM ×2500).

Despite continuous fluid therapy, the patient's levels of BUN and Cr increased gradually, and thus renal biopsy was performed on day 3 of admission. The kidney biopsy showed acute tubular necrosis without infiltration of inflammatory cells. Although the histologic findings were not diffuse, definite features of tubular damage were noted. Necrotic sloughing of tubular cells was found, with mitotic figures present in regenerating tubular cells. Necrotic cellular casts were also noted in the tubular lumina (Figure 1). Ultrastructural examination revealed electron-dense deposits in subendothelium of glomerular capillaries. And focal effacement and fusion of foot processes of podocytes were noted (Figure 2). Tubular cells contained electron-dense lysosomes in the cytoplasm and lumina (Figure 3).

The development of acute respiratory distress syndrome was observed on day 4: arterial PaO_2_/FiO_2_ < 100 mmHg. Corticosteroid therapy for acute lung injury was administered for 28 days, beginning on day 5 (1 mg/kg/day of intravenous methylprednisolone for 2 weeks, followed by a 2-week taper). Hemodialysis was provided 5 times from day 5 to day 10 for the treatment of hyperkalemia and oliguria. After the termination of hemodialysis, the patient's renal function recovered completely. During the administration of corticosteroid therapy, pulmonary fibrosis was observed on chest computed tomography. On day 16, bilateral spontaneous pneumothorax, pneumomediastinum, and subcutaneous emphysema occurred. Although bilateral closed tube thoracotomy and a lung protective ventilation strategy were applied, the pneumothorax and pulmonary fibrosis progressed gradually. The patient died of complicated lung fibrosis and persistent pneumothorax/pneumomediastinum on day 40 of hospitalization.

## 3. Discussion

In the present case, the patient demonstrated AKI and mild proteinuria after ingesting 50 g of silica-containing fertilizer. This amount might be toxic because the upper safety level for a 70 kg human man is 1,750 mg/day [3]. Acute kidney injury due to silica exposure is rare. In one reported case, a 23-year-old sandblaster showed massive proteinuria, AKI, and acute pulmonary silicoproteinosis [4]. His kidneys showed mild proliferative glomerulonephritis with granular deposits of immunoglobulin M and complement protein C3 along the glomerular basement membrane. The other case involved a 54-year-old man who demonstrated mucosal damage in the upper gastrointestinal tract and oliguric renal failure after 135 g of silicon ingestion. He recovered after 8 sessions of hemodialysis and did not undergo kidney biopsy [5].

Silica nephropathy usually occurs after heavy, long-term exposure to silica dust for a long period. It initially manifests as low urinary specific gravity, proteinuria, hypertension, and subsequent progressive decline in glomerular filtration rate [6]. Occupational exposures confer a higher risk of CKD. In a cohort study of silica-exposed gold miners, the incidence of ESRD was increased 7.7-fold among workers with more than 10 years of exposure [7]. In 4,626 silica-exposed industrial sand workers, the ESRD incidence was 7.79 times greater among those in the highest quartile of cumulative exposure [8]. In addition, 10% of 583 individuals with silicosis had suspected CKD [9]. Any silica exposure was related to a 40% increased risk of CKD [10]. Rapidly progressive glomerulonephritis was also reported in patients with chronic exposure [11].

Silica nephropathy is also related to systemic autoimmune diseases, including systemic lupus erythematosus, Goodpasture's syndrome, scleroderma, polyarteritis nodosa, and, in particular, c-ANCA-positive Wegener's granulomatosis [2]. Furthermore, approximately 25−50% of patients with pulmonary silicosis might demonstrate elevated ANA titers [12]. Our patient presented with severe pulmonary fibrosis and a low ANA titer. It is not clear whether his ANA level increased during the period of treatment for pulmonary fibrosis, as we did not reexamine the ANA titer.

In case of chronic exposure, there is no single pathologic finding of silica nephropathy; its varied pathology includes focal glomerulonephritis, intraluminal sloughing of the proximal tubule, proliferative glomerulonephritis, and crescentic glomerulonephritis [4, 13–15]. Electron microscopy revealed foot process obliteration, characteristic cytoplasmic dense lysosome, microtubule, and dense deposit. However, little is known what pathologic finding is in acute exposure. In animal study, silicon was direct toxic to kidney, causing acute tubular necrosis [16]. Our patient showed only acute tubular necrosis without evidence of inflammatory cell infiltration. Our case also revealed subendothelial electron-dense deposits with foot process loss in the glomerular capillaries. Electron-dense lysosomes were also found in the proximal tubules. These ultrastructural findings are compatible with previous reports [11].

The treatment for silica nephropathy has not yet been established. The main goal is the removal of the source of silica exposure in order to minimize disease progression. The treatment course depends on the mechanism and stage of the disease and ranges from blood pressure control to administration of steroids or cytotoxic agents. It remains unclear whether the cause of the current patient's renal recovery was the effects of the steroid or spontaneous remission, as steroid treatment was conducted to treat his pulmonary fibrosis.

In conclusion, we report a case of sodium silicate poisoning that resulted in AKI with acute tubular necrosis requiring hemodialysis. As acute intoxication can occur and no antidote or specific therapy exists, prevention of accidental or intentional silica ingestion using careful packaging and warnings is critical.
